# Supplementary material for: Design of a methotrexate-controlled chemical dimerization system and its use in bio-electronic devices
Source: Nat Commun. 2021 Dec 8;12:7137. doi: 10.1038/s41467-021-27184-w (PMC8654847; doi:10.1038/s41467-021-27184-w)
Supplement: Supplementary file 1 — Supplementary Information [file 41467_2021_27184_MOESM1_ESM.pdf]

Supplementary Information

Design of a methotrexate-controlled chemical dimerization system and its use in bio-electronic devices

Zhong Guo, Oleh Smutok, Wayne A. Johnston, Patricia Walden, Jacobus P. J. Ungerer, Thomas S. Peat, Janet Newman, Jake Parker, Tom Nebl, Caryn Hepburn, Artem Melman, Richard J. Suderman, Evgeny Katz and Kirill Alexandrov

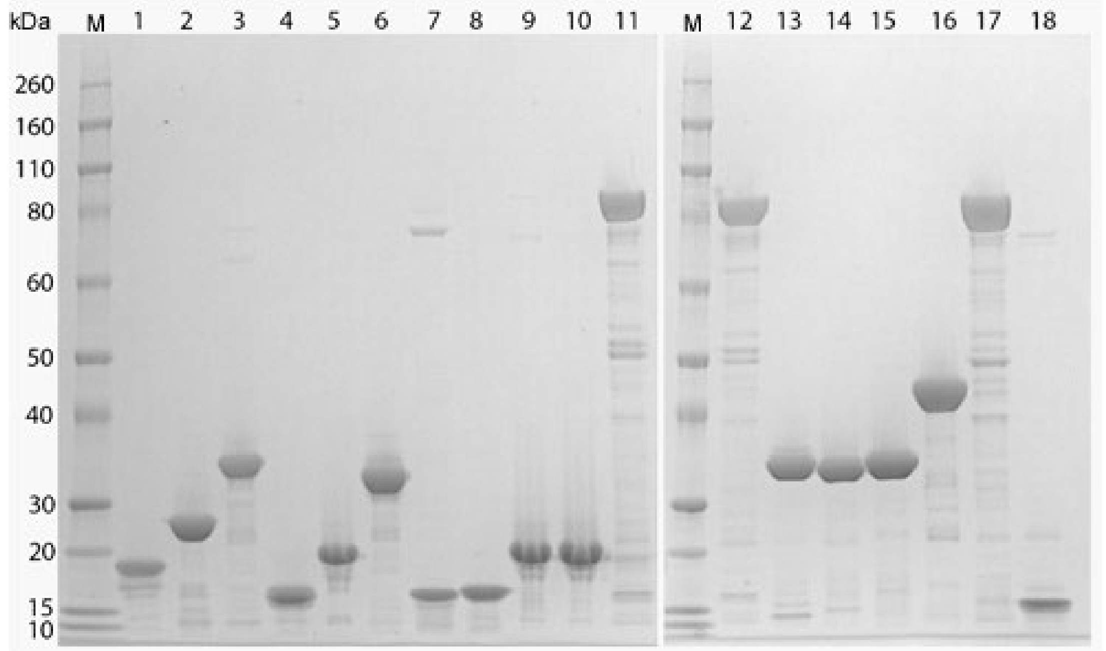

Figure S1. *SDS-PAGE analysis of recombinant and biotinylated proteins used in this study.* Approximately 0.5 μg of protein was loaded onto 15% SGS-PAGE gel and upon electrophoresis stained with Coomassie Brilliant Blue and photographed in visible light. The weights in kDa of the molecular weight markers (M) are indicated on the left hand side.(1) VHH-Biotin AVI, (2) DHFR-Biotin AVI, (3) Biotin AVI-Thymidylate Synthase, (4) VHH, (5) DHFR, (6) Thymidylate Synthase, (7) nanoCLAMP5, (8) nanoCLAMP8, (9) nanoCLAMP5-CaM-BP, (10) nanoCLAMP8-CaM-BP, (11) GDH-CaM-VHH, (12) GDH-CaM-VHH, (13) nanoCLAMP3-VHH, (14) nanoCLAMP5-VHH, (15) nanoCLAMP8-VHH, (16) EGFP-VHH, (17) GDH-CaM-FKBP, (18) FRB-CaM-BP.

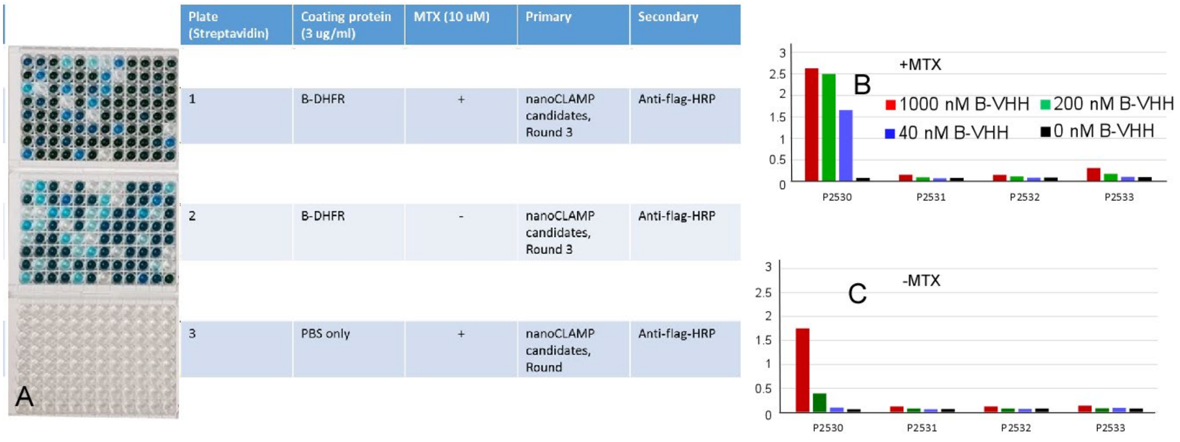

Plate (Streptavidin)	Coating protein (3 ug/ml)	MTX (10 uM)	Primary	Secondary
1	B-DHFR	+	nanoCLAMP candidates, Round 3	Anti-flag-HRP
2	B-DHFR	-	nanoCLAMP candidates, Round 3	Anti-flag-HRP
3	PBS only	+	nanoCLAMP candidates, Round	Anti-flag-HRP

Figure S2*.* Analysis of the nanoCLAMP clones selected against DHFR:MTX complex (A) *semELISA of individual clones from panning round 3 of phage display library C11 vs. biotinylated DHFR.* Wells were coated with biotinylated protein at 3 μg/ml or PBS as negative control and blocked with 2% dry milk in PBS-T (0.05% Tween 20). *E. coli* expression culture media containing candidate secreted flag-tagged nanoCLAMPs was used as primary. The primary was detected with anti-flag-HRP (Sigma A8592), and developed with TMB Ultra (Thermo 34028). Primary and secondary proteins were diluted in blocking buffer. Positives (qualitative) are A1, C1, E1, G2, A4, A6, A8, F8, E10, C11, G11, G12. (B) Inverse ELISA of recombinant purified nanoCLAMPs clones p2530 (well A1 in A) ,p2531(well A8), p2532(well A4), p2533 (well C1) assessed for binding to DHFR in the presence of indicated concentrations of MTX. Maleimide coated plates were coated with purified nanoCLAMPs and incubated with three concentrations of biotinylated target protein and 10 μM MTX. The binding was detected using a Streptavidin-HRP mediated colorimetric reaction. (C) as in B but without MTX.

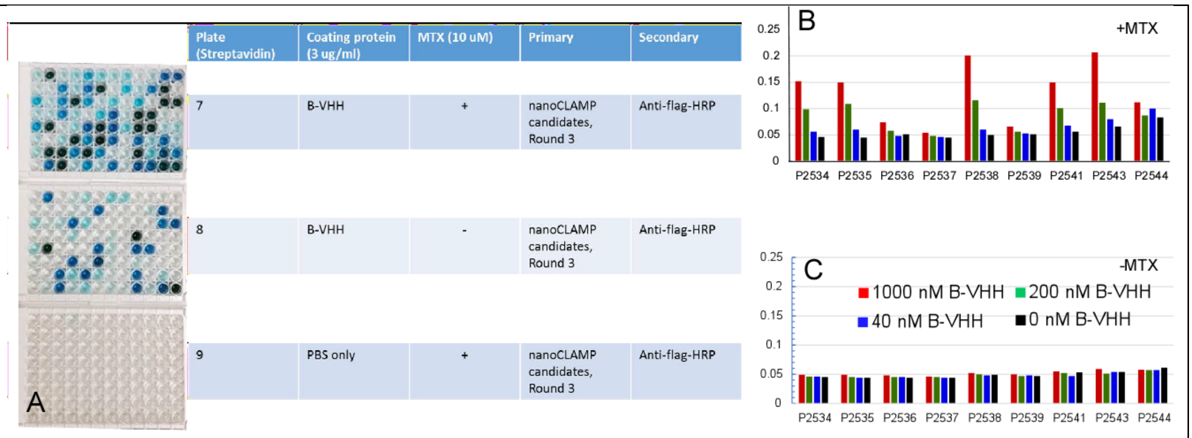

Figure S3. Analysis of the nanoCLAMP clones selected against VHH:MTX complex (A) semELISA of individual clones from panning round 3 of phage display library C11 vs. biotinylated anti-MTX VHH . Wells were coated with biotinylated protein at 3 μg/ml or PBS as negative control and blocked with 2% dry milk in PBS-T (0.05% Tween 20). *E. coli* expression culture media containing candidate secreted flag-tagged nanoCLAMPs was used as primary. The primary was detected with anti-flag-HRP (Sigma A8592), and developed with TMB Ultra (Thermo 34028). Primary and secondary proteins were diluted in blocking buffer. Positives (qualitative) are G2(clone4), H2(clone2), B3, D3, B5, E5, F5(clone6), D6 (clone1), C7(clone7), H7(clone5), B8, E8(clone8), C9, F9, B10, D10(clone9), H10, A11, F11(clone3), A12, G12. (B) Inverse ELISA of recombinant purified nanoCLAMPs clones p2534 (well D-6) ,p2535(well H2), p2536(well F11), p2537(well G2), p2538 (well H7), p2539 (well F5), p2541 (well C7), p2543(well E8), p2544 (well D10) assessed for binding to VHH in the presence of indicated concentrations of MTX. Maleimide coated plates were coated with purified nanoCLAMPs and incubated with three concentrations of biotinylated target protein and 10 μM MTX. The binding was detected using a Streptavidin-HRP mediated colorimetric reaction. (C) as in B but without MTX.

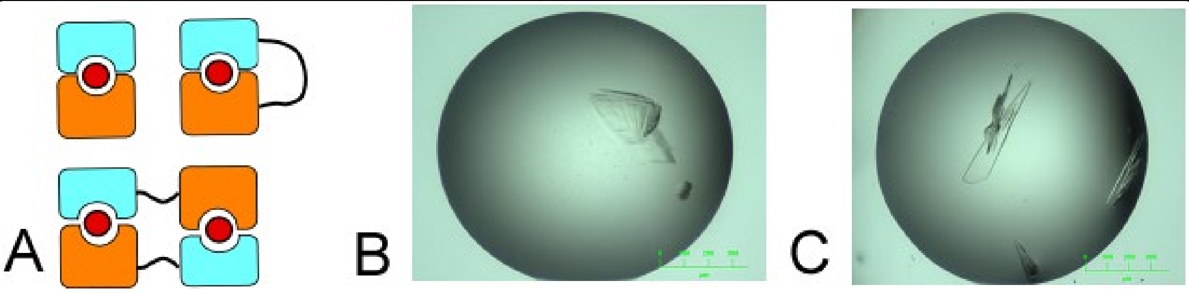

Figure S4. Crystallization of MTX:VHH:nanoCLAMP complexes. (A) Schematic of non-covalent or covalent MTX:VHH:nanoCLAMP complexes produced for crystallization trials (Table S1). (B) An example of the crystals obtained for MTX:VHH:nanoCLAMP clone 3 complex (C) An

example of the crystals obtained for MTX:VHH:nanoCLAMP clone 8 complex. The pictures were taken once.

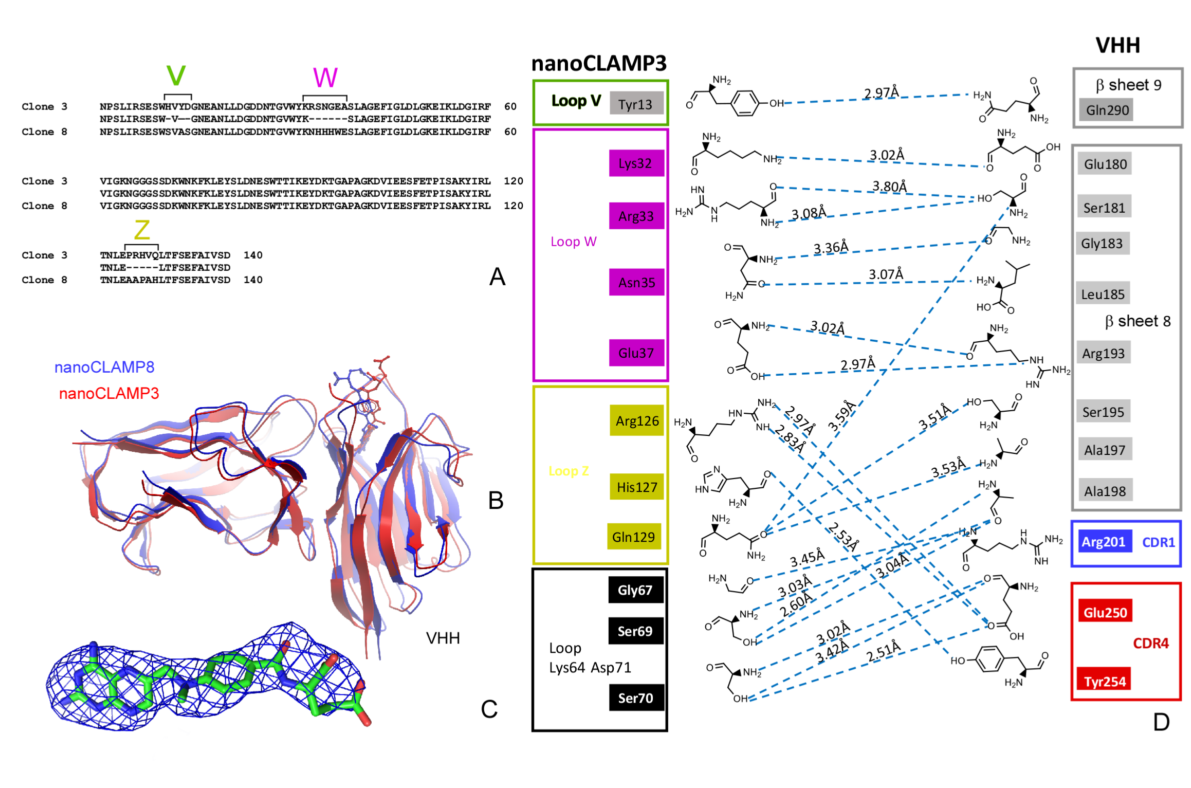

Figure S5. *Structure of MTX:VHH:nanoCLAMP3 complex.* (A) Sequence alignment of NanoCalmp3 and NanoCLAMP8. The Loop V, W and Z were highlighted. (B) Superimposition of the structure of MTX:VHH in complex with NanoCalmp8 and nanoCLAMP3. (C) OMIT map of MTX of MTX:VHH:nanoCLAMP3 structure contoured at 3 σ. (D) graphic representation of the interface between nanoCLAMP3 and VHH. The hydrogen bonds are shown as blue dashed lines and their lengths are displayed as numbers.

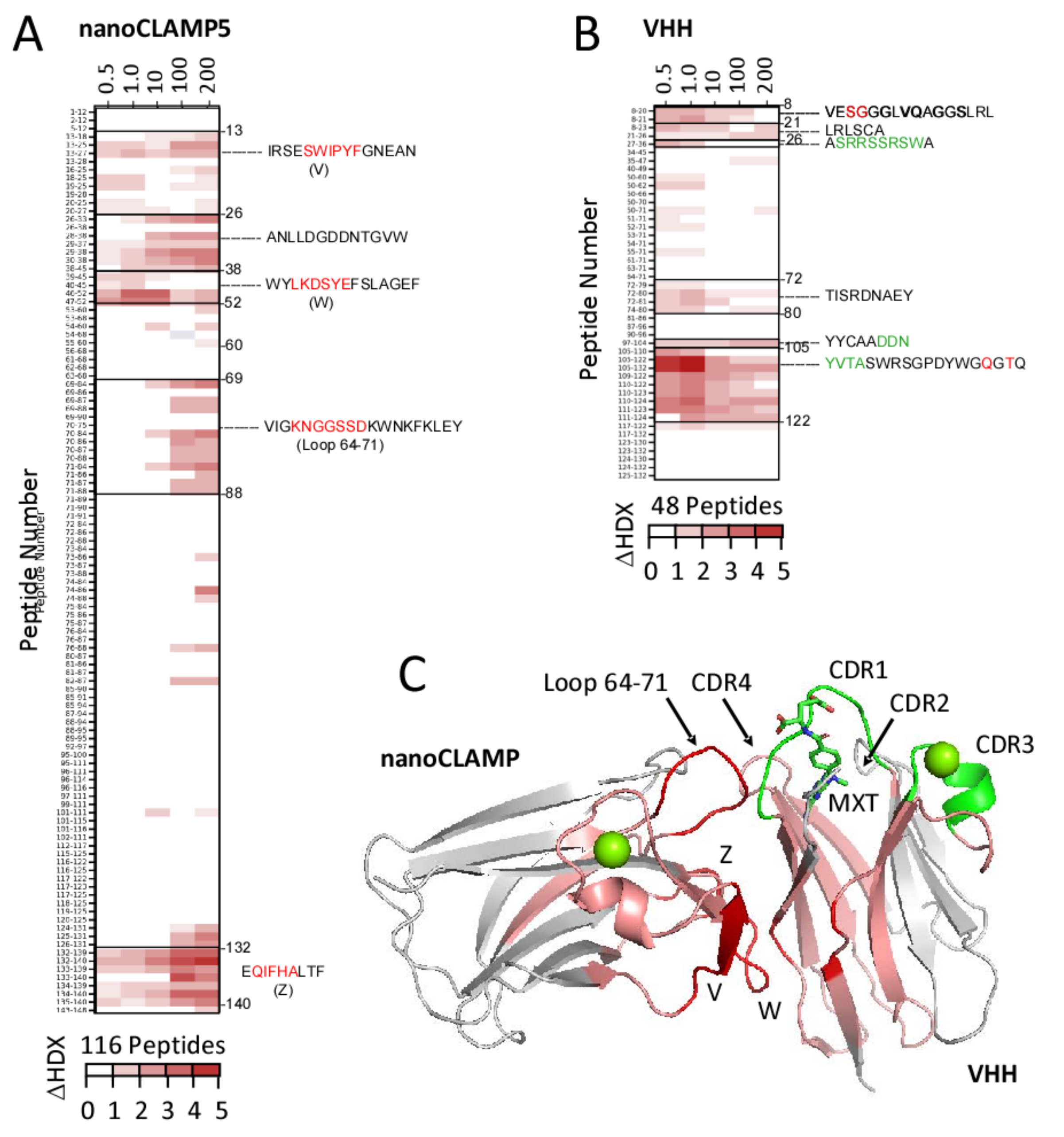

Figure S6. *HDX analysis of HTX:VHH:nanoCLAMP5 complex.* Comparative HDX-MS analyses of nanoCLAMP5 and VHH in the presence or absence of methotrexate suggest that similar parts of the molecules are involved in nanoCLAMP complex formation. Chiclet plots show differences in deuterium uptake of VHH:NC5 complexes in the absence MTX – deuterium exchange of peptic peptides in the presence of MTX (delta HDX) for nanoCLAMP5 (A) or VHH (B). Experiments were carried out in triplicate and differences I deuterium uptake that have a p-value > 0.01 are shown as zero difference (white). Peptide sequences of regions showing significant protection after 0.5, 1.0, 10, 100, 200 minutes exposure are shown (red shades). Sequences involved in nanoCLAMP complex formation are highlighted in red and

mapped onto the X-ray crystal structure of MTX:VHH:nanoCLAMP8 (C). CDR sequences involved in MTX ligand binding are highlighted in green.

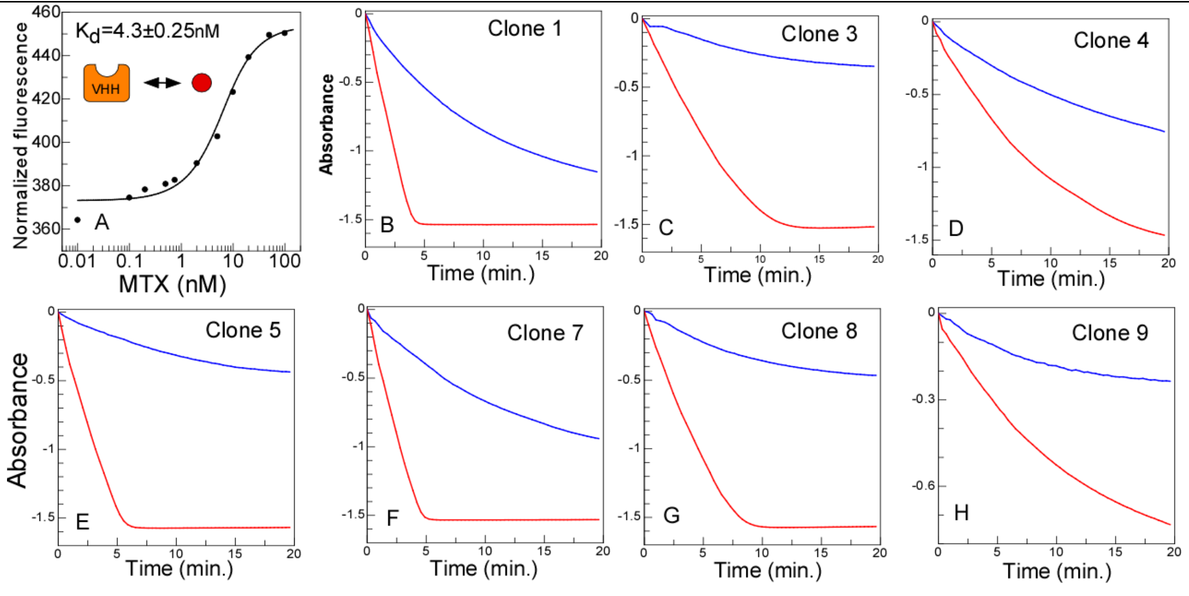

Figure S7. Interaction analysis of VHH and anti-MTX anoCLAMP and activity analysis of two component MTX biosensors based on nanoCLAMP:VHH CID system. (A) MST titration of 10 nM of EGFP-VHH with increasing concentrations of MTX leading to a K_d fit of 4.3±0.25 nM. (B-F) Time resolved changes in absorbance of DCPIP as a measure of GDH enzymatic GDH activity of 10 nM VHH-GDH-CaM and 100 nM nanoCLAMP-CaM-BP fusion proteins (clone 1-9) was measured in the presence and in the absence of 1 μM MTX in the buffer containing 20 mM Tris-HCl, pH 7.2, 100 mM NaCl, 1 mM $CaCl_2$.

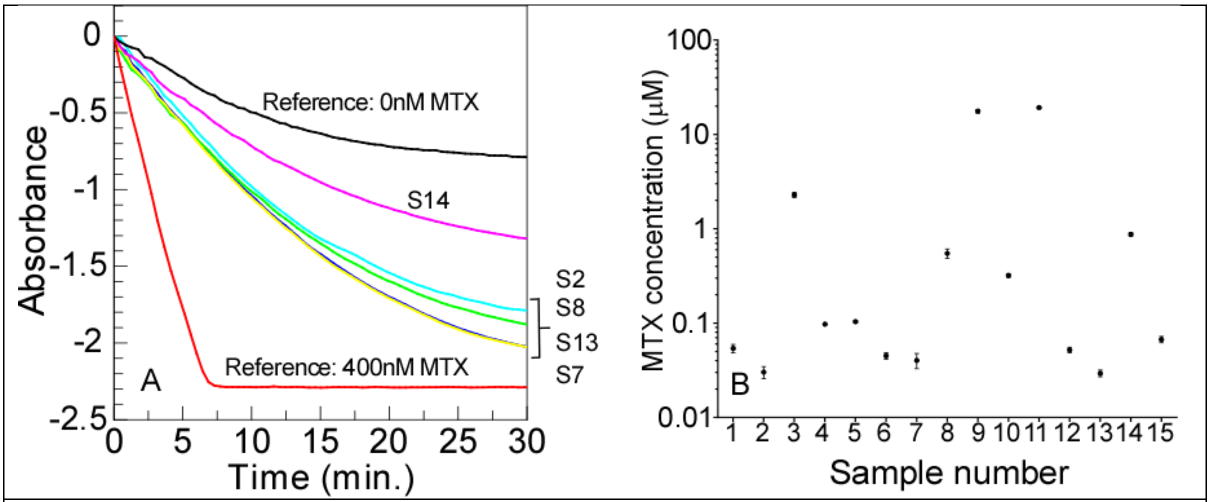

Figure S8. Quantification of MTX in the patient serum using the GDH biosensor-based assay. (A) Representative time traces of the absorbance changes in the assay reactions supplemented with 1:40 diluted patient or reference serum. The concentration of the reference reflects the concentration of the drug in undiluted serum. The fit of the initial rates of sample 14 to the calibration curve results in the calculated serum concentration of 10nM. The samples 2,7,8,13 were calculated to contain MTX at concentration close to 50nM. Minor diversion of traces at the end of the reaction is not accounted for as only the fit of the initial rates were used for calculations. (B) A plot of MTX concentrations in samples of patients undergoing MTX therapy determined using the assay based on MTX-biosensor. The average of three experiments were plotted with error bars reflecting standard deviation of the data. The center of the data represents the mean of the data for each individual sample. In some cases, the error bars could not be displayed due to their small sizes.

Fig.S9

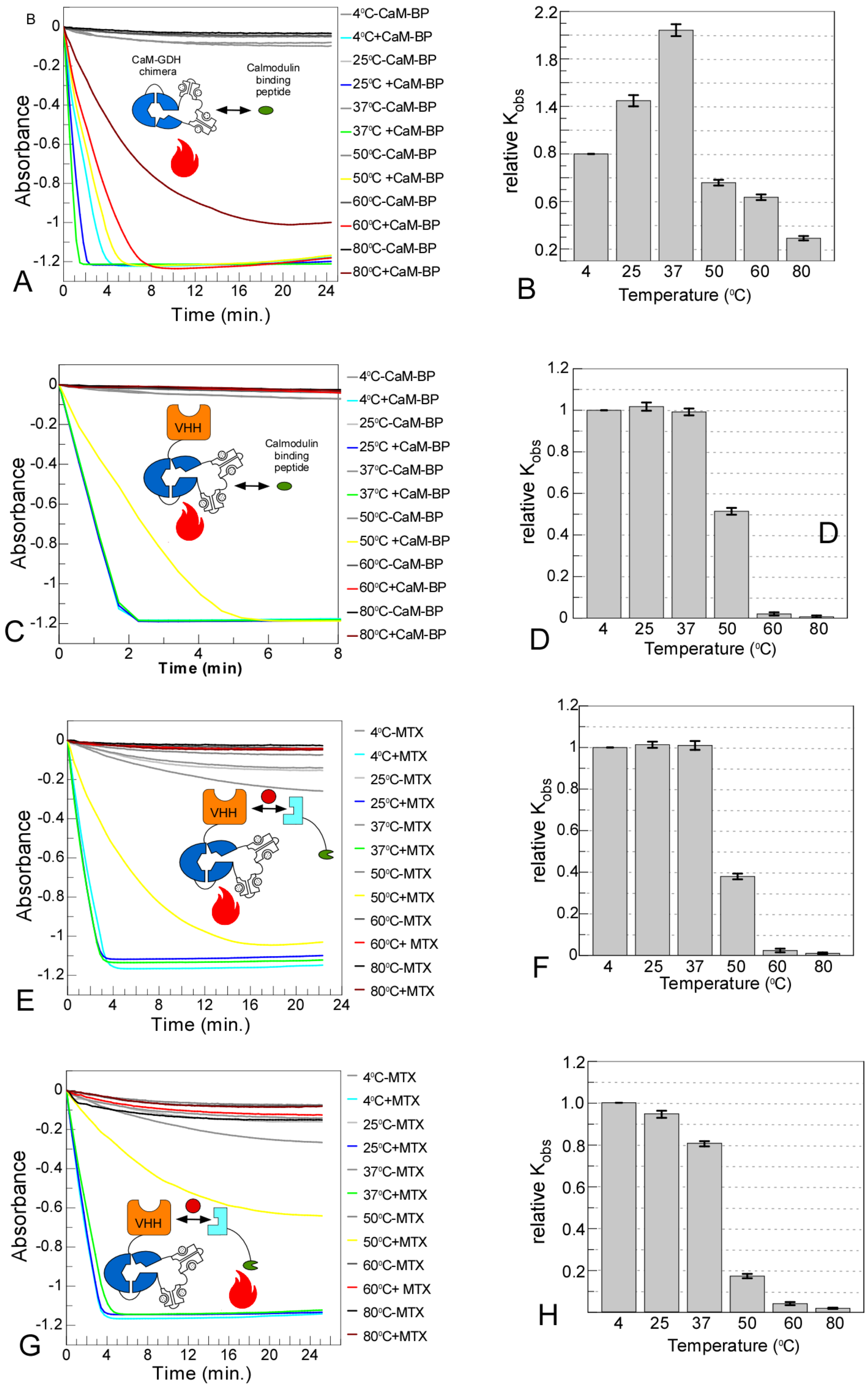

Figure S9. Thermostability analysis of GDH-based biosensors. (A) Changes in absorbance of DCPIP as a measure of GDH enzymatic activity of 10nM solution of GDH-CaM fusion

preincubated at indicated temperatures prior to addition of 1μM CaM-BP. The flame icon marks the molecules subjected to thermostability analysis. The experiments were performed in triplicates. (B) A bar plot of normalized GDH activity derived from A. The bars represent values of average of three independent measurements performed in the same experiment. The error bars denote positive and negative boundaries of the standard error of mean. (C) As in A but using solution of 10nM VHH-GDH-CaM fusion and 1μM CaM-BP. The experiments were performed in triplicates. (D) A bar plot of normalized GDH activity derived from C. (E) Changes in absorbance of DCPIP as a measure of GDH enzymatic activity of 10nM solution of VHH-GDH-CaM fusion pre-incubated at indicated temperatures and mixed with 100nM of fresh nanoCLAMP-CaM-BP and 100nM MTX where indicated. The experiments were performed in triplicates. (F) A bar plot of normalized GDH activity derived from E. (G) As in E but using 100nM of nanoCLAMP-CaM-BP that was pretreated at various temperatures, fresh 10nM solution of VHH-GDH-CaM fusion and 100nM MTX where indicated. (G) A bar plot of normalized GDH activity derived from E. In all plots the bars represent values of average of three independent measurements performed in the same experiment. The error bars denote positive and negative boundaries of the standard error of mean.

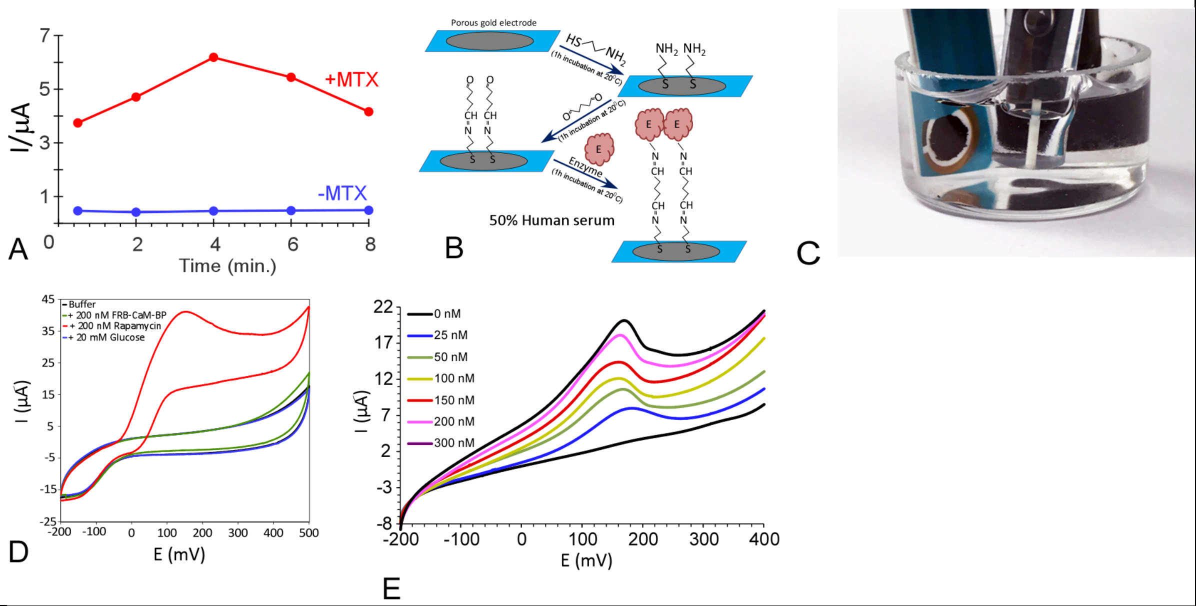

Figure S10. *Electrochemical analysis of two component MTX biosensor.* (A) Time resolved activity of solution-based two component MTX assay in the presence or absence of the ligand. The decay in signal after 4 min reflects precipitation of mPMS electron mediator. The plot represents results of individual measurements. (B) Principal scheme of the

immobilization of the GDH component of the MTX biosensor on the working electrode surface. (C) A photograph of the cell used to carry out electrochemical analysis of electrodes with immobilized two component biosensors. (D) A typical cyclic voltammograms for Rapamycin biosensor-based bioelectrode: black- buffer solution (25 mM Tris-H_2SO_4 buffer, pH 7.2, 100 mM Na_2SO_4 and 1 mM $Ca(CH_3COO)_2$); blue- in presence of 20 mM glucose; green- in presence of 20 mM glucose and 200 nM FRB-CaM-BP; red- in presence of 20 mM glucose, 200 nM FRB-CaM-BP and 200 nM Rapamycin. The electrode was scanned at the rate of 2 mV/s *vs*. Ag/AgCl/3 M KCl reference electrode at room temperature. (E) Analysis of MTX biosensor-based bioelectrode in 50% human serum. The serum was diluted to a final concentration of the following buffer 25 mM Tris-H_2SO_4 buffer, pH 7.2, 100 mM Na_2SO_4, 1 mM $Ca(CH_3COO)_2$, 20 mM glucose and 200 nM nanoCLAMP-CaM-BP. The electrode was scanned at the rate of 2 mV/s *vs*. Ag/AgCl/3M KCl reference electrode at room temperature. The figure represents a typical cyclic voltammogram.

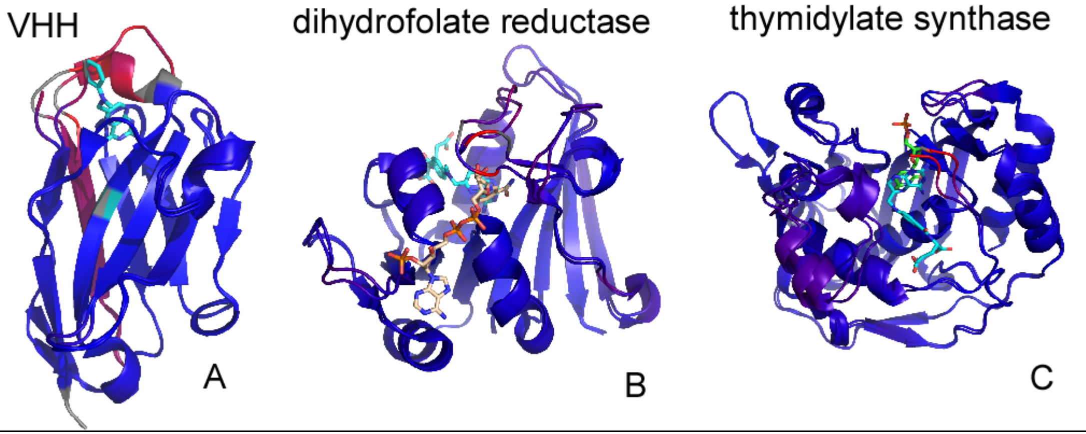

Figure S11. *Analysis of the conformational changes in MTX:protein complexes.* Structural data of apo- and holo- bound proteins was collected from the Protein Data Bank for DHFR (apo: 4EIZ, holo: 3DAU); thymidylate synthase (apo: 1F4B, holo: 1AXW); and anti-methotrexate VHH (apo: 3qxw, while the biosensor complex was used as the holo structure). RMSD was visualized using the colorbyRMSD script in Pymol, which aligns two structures, and colors the residues by the distance between their C-alpha atoms with blue representing the minimum distance and red the maximum.

Supplementary Table 1. Summary of protein sequences used in this study. The sequences are colored according to the functional elements.

dihydrofolate reductase - AVI tag	MDMISLIAALAVDRVIGMENAMPWNLPADLAWFKRNTLNKPVIMGRHTWESIGRPLPGRKNIILSSQPGTDDRVTWVKSVDEAIAACGDVPEIMVIGGGRVYEQFLPKAQKLYLTHIDAEVEGDTHFPDYEPDDWESVFSEFHDADAQNSHSYCFEILERRGSGLEVLFQGPGSGSGGLNDIFEAQKIEWHEKLAAALEHHHHHH
dihydrofolate reductase	MDMISLIAALAVDRVIGMENAMPWNLPADLAWFKRNTLNKPVIMGRHTWESIGRPLPGRKNIILSSQPGTDDRVTWVKSVDEAIAACGDVPEIMVIGGGRVYEQFLPKAQKLYLTHIDAEVEGDTHFPDYEPDDWESVFSEFHDADAQNSHSYCFEILERRGKLAAALEHHHHHH
AVI tag-thymidylate synthase	MDGHHHHHHGSGGLNDIFEAQKIEWHEGSGSGLEVLFQGPGSGMKQYLELMQKVLDEGTQKNDRTGTGTLSIFGHQMRFNLQDGFPLVTTKRCHLRSIIHELLWFLQGDTNIAYLHENNVTIWDEWADENGDLGPVYGKQWRAWPTPDGRHIDQITTVLNQLKNDPDSRRIIVSAWNVGELDKMALAPCHAFFQFYVADGKLSCQLYQRSCDVFLGLPFNIASYALLVHMMAQQCDLEVGDFVWTGGDTHLYSNHMDQTHLQLSREPRPLPKLIIKRKPESIFDYRFEDFEIEGYDPHPGIKAPVAI
thymidylate synthase	MGHHHHHHGSGMKQYLELMQKVLDEGTQKNDRTGTGTLSIFGHQMRFNLQDGFPLVTTKRCHLRSIIHELLWFLQGDTNIAYLHENNVTIWDEWADENGDLGPVYGKQWRAWPTPDGRHIDQITTVLNQLKNDPDSRRIIVSAWNVGELDKMALAPCHAFFQFYVADGKLSCQLYQRSCDVFLGLPFNIASYALLVHMMAQQCDLEVGDFVWTGGDTHLYSNHMDQTHLQLSREPRPLPKLIIKRKPESIFDYRFEDFEIEGYDPHPGIKAPVAI
VHH-AVI tag	MDGSQVQLVESGGGLVQAGGSLRLSCAASRRSSRSWAMAWFRQAPGKEREFVAKISGDGRLTTYGDSVKGRFTISRDNAEYLVYLQMDSLKPEDTAVYYCAADDNYVTASWRSGPDYWGQGTQVTVSSGSGLEVLFQGPGSGSGGLNDIFEAQKIEWHEKLAAALEHHHHHH
VHH	MDGSQVQLVESGGGLVQAGGSLRLSCAASRRSSRSWAMAWFRQAPGKEREFVAKISGDGRLTTYGDSVKGRFTISRDNAEYLVYLQMDSLKPEDTAVYYCAADDNYVTASWRSGPDYWGQGTQVTVSSGKLAAALEHHHHHH
nanoCLAMP1-GS linker-Cys P2534	MGSSHHHHHHNPSLIRSESWAAIIGNEANLLDGDDNTGVWYKKNGDEKSLAGEFIGLDLGKEIKLDGIRFVIGKNGGGSSDKWNKFKLEYSLDNESWTTIKEYDKTGAPAGKDVIEESFETPISAKYIRLTNLEQRSLALTFSEFAIVSDGGGGSGGGGSGGGC
nanoCLAMP2-GS linker-Cys P2535	MGSSHHHHHHNPSLIRSESWAAYYGNEANLLDGDDNTGVWYYNATSSYSLAGEFIGLDLGKEIKLDGIRFVIGKNGGGSSDKWNKFKLEYSLDNESWTTIKEYDKTGAPAGKDVIEESFETPISAKYIRLTNLESKTFNLTFSEFAIVSDGGGGSGGGGSGGGC
nanoCLAMP3-GS linker-Cys P2536	MGSSHHHHHHNPSLIRSESWHVYDGNEANLLDGDDNTGVWYKRSNGEASLAGEFIGLDLGKEIKLDGIRFVIGKNGGGSSDKWNKFKLEYSLDNESWTTIKEYDKTGAPAGKDVIEESFETPISAKYIRLTNLEPRHVQLTFSEFAIVSDGGGGSGGGGSGGGC
nanoCLAMP4-GS linker-Cys P2537	MGSSHHHHHHNPSLIRSESWHVYDGNEANLLDGDDNTGVWYGKYSFHKSLAGEFIGLDLGKEIKLDGIRFVIGKNGGGSSDKWNKFKLEYSLDNESWTTIKEYDKTGAPAGKDVIEESFETPISAKYIRLTNLEHKESLLTFSEFAIVSDGGGGSGGGGSGGGC

nanoCLAMP5-GS linker-Cys P2538	MGSSHHHHHHNPSLIRSESWIPYFGNEANLLDGDDNTGVWYLKDSYEFSLAGEFIGLDLGKEIKLDGIRFVIGKNGGGSSDKWNKFKLEYSLDNESWTTIKEYDKTGAPAGKDVIEESFETPISAKYIRLTNLEQIFHALTFSEFAIVSDGGGGSGGGGSGGGC
nanoCLAMP6-GS linker-Cys P2539	MGSSHHHHHHNPSLIRSESWSFSYGNEANLLDGDDNTGVWYDVKASTSSLAGEFIGLDLGKEIKLDGIRFVIGKNGGGSSDKWNKFKLEYSLDNESWTTIKEYDKTGAPAGKDVIEESFETPISAKYIRLTNLEPAYEFLTFSEFAIVSDGGGGSGGGGSGGGC
nanoCLAMP7-GS linker-Cys P2541	MGSSHHHHHHNPSLIRSESWSPAVGNEANLLDGDDNTGVWYKKYGNSESLAGEFIGLDLGKEIKLDGIRFVIGKNGGGSSDKWNKFKLEYSLDNESWTTIKEYDKTGAPAGKDVIEESFETPISAKYIRLTNLENAERALTFSEFAIVSDGGGGSGGGGSGGGC
nanoCLAMP8-GS linker-Cys P2543	MGSSHHHHHHNPSLIRSESWSVASGNEANLLDGDDNTGVWYKNHHHWESLAGEFIGLDLGKEIKLDGIRFVIGKNGGGSSDKWNKFKLEYSLDNESWTTIKEYDKTGAPAGKDVIEESFETPISAKYIRLTNLEAAPAHLTFSEFAIVSDGGGGSGGGGSGGGC
nanoCLAMP9-GS linker-Cys P2544	MGSSHHHHHHNPSLIRSESWVPFIGNEANLLDGDDNTGVWYVKSGLNGYSLAGEFIGLDLGKEIKLDGIRFVIGKNGGGSSDKWNKFKLEYSLDNESWTTIKEYDKTGAPAGKDVIEESFETPISAKYIRLTNLEYTFFVLTFSEFAIVSDGGGGSCGGGSGGGC
nanoCLAMP5	MDSHHHHHHNPSLIRSESWIPYFGNEANLLDGDDNTGVWYLKDSYEFSLAGEFIGLDLGKEIKLDGIRFVIGKNGGGSSDKWNKFKLEYSLDNESWTTIKEYDKTGAPAGKDVIEESFETPISAKYIRLTNLEQIFHALTFSEFAIVSD
nanoCLAMP8	MDSHHHHHHNPSLIRSESWSVASGNEANLLDGDDNTGVWYKNHHHWESLAGEFIGLDLGKEIKLDGIRFVIGKNGGGSSDKWNKFKLEYSLDNESWTTIKEYDKTGAPAGKDVIEESFETPISAKYIRLTNLEAAPAHLTFSEFAIVSD
nanoCLAMP5-CaM BP	MDGSSHHHHHHNPSLIRSESWIPYFGNEANLLDGDDNTGVWYLKDSYEFSLAGEFIGLDLGKEIKLDGIRFVIGKNGGGSSDKWNKFKLEYSLDNESWTTIKEYDKTGAPAGKDVIEESFETPISAKYIRLTNLEQIFHALTFSEFAIVSDGGSGSGGSGGGSGSSGGSGGSGGGKRRWKKNFIAVSAANR
nanoCLAMP8-CaM BP	MDGSSHHHHHHNPSLIRSESWSVASGNEANLLDGDDNTGVWYKNHHHWESLAGEFIGLDLGKEIKLDGIRFVIGKNGGGSSDKWNKFKLEYSLDNESWTTIKEYDKTGAPAGKDVIEESFETPISAKYIRLTNLEAAPAHLTFSEFAIVSDGGSGSGGGGGSGSSGGSGGSGGGKRRWKKNFIAVSAANR
nanoCLAMP3-VHH	MDHHHHHHSNPSLIRSESWHVYDGNEANLLDGDDNTGVWYKRSNGEASLAGEFIGLDLGKEIKLDGIRFVIGKNGGGSSDKWNKFKLEYSLDNESWTTIKEYDKTGAPAGKDVIEESFETPISAKYIRLTNLEPRHVQLTFSEFAIVSDGGSGSGASGSGSGSGSGASGGSSGGSGGSGGGGSQVQLVESGGGLVQAGGSLRLSCAASRRSSRSWAMAWFRQAPGKEREFVAKISGDGRLTTYGDSVKGRFTISRDNAEYLVYLQMDSLKPEDTAVYYCAADDNYVTASWRSGPDYWGQGTQVTVSS
nanoCLAMP5-VHH	MDHHHHHHSNPSLIRSESWIPYFGNEANLLDGDDNTGVWYLKDSYEFSLAGEFIGLDLGKEIKLDGIRFVIGKNGGGSSDKWNKFKLEYSLDNESWTTIKEYDKTGAPAGKDVIEESFETPISAKYIRLTNLEQIFHALTFSEFAIVSDGGSGSGASGSGSGSGSGASGGSSGGSGGSGGGGSQVQLVESGGGLVQAGGSLRLSCAASRRSSRSWAMAWFRQAPGKEREFVAKISGDGRLTTY

	GDSVKGRFTISRDNAEYLVYLQMDSLKPEDTAVYYCAADDNYVTASWR SGPDYWGQGTQVTVSS
nanoCLAMP8-VHH	DHHHHHHSNPSLIRSESWSVASGNEANLLDGDDNTGVWYKNHHHWE SLAGEFIGLDLGKEIKLDGIRFVIGKNGGGSSDKWNKFKLEYSLDNESW TTIKEYDKTGAPAGKDVIEESFETPISAKYIRLTNLEAAPAHLTFSEFAIVS DGGSGSGASGSGSGSGSGASGGSSGGSGGSGGGSQVQLVESGGGLV QAGGSLRLSCAASRRSSRSWAMAWFRQAPGKEREFVAKISGDGRLTTY GDSVKGRFTISRDNAEYLVYLQMDSLKPEDTAVYYCAADDNYVTASWR SGPDYWGQGTQVTVSS
EGFP-VHH	MDGVSKGEELFTGVVPILVELDGDVNGHKFSVSGEGEGDATYGKLTLKF ICTTGKLPVPWPTLVTTLGYGVQCFSRYPDHMKQHDFFKSAMPEGYVQ ERTIFFKDDGNYKTRAEVKFEGDTLVNRIELKGIDFKEDGNILGHKLEYN YNSHNVYIMADKQKNGIKVNFKIRHNIEDGSVQLADHYQQNTPIGDGP VLLPDNHYLSTQSALSKDPNEKRDHMVLLEFVTAAGITLGMDELYKGGS GGGSQVQLVESGGGLVQAGGSLRLSCAASRRSSRSWAMAWFRQAPGK EREFVAKISGDGRLTTYGDSVKGRFTISRDNAEYLVYLQMDSLKPEDTA VYYCAADDNYVTASWRSGPDYWGQGTQVTVSSKLAAALEHHHHHH
VHH-GDH-CaM	DVPLIPSQFAKAKSENFDKKVILSNLNKPHALLWGPDNQIWLTERATGK ILRVNPESGSVKTVFQVPEIVNDADGQNGLLGFAFHPDFKNNPYIYISGT FKNPKSTDKELPNQTIIRRYTYNKSTDTLEKPVDLLAGLPSSKDHQSGRL VIGPDQKIYYTIGDQGRNQLAYLFLPNQAQHTPTQQELNGKDYHTYMG KVLRLNLDGSIPKDNPSFNGVVSHIYTLGHRNPQGLAFTPNGKLLQSEQ GPNSDDEINLIVKGGNYGWPNVAGYKDDSGYAYANYSAAANKTIKDLA QNGVKVAAGVPVTKESEWTGKNFVPPLKTLYTVQDTYNYNDPTCGEMT YICWPTVAPSSAYVYKGGKKAITGWENTLLVPSLKRGVIFRIKLDPTYST TYDDAVPMFKSGSGGTEEQIAEFKEAFSLFDKDGDGTITTKELGTVMRS LGQNPTEAELQDMINEVDADGNGTIDFPEFLTMMARKMKDTDSEEEIR EAFRVFDKDGNGYISAAELRHVMTNLGEKLTDEEVDEMIREADIDGDG QVNYEEFVQMMTAGGSSGNRYRDVIASPDGNVLYVLTDTAGNVQKDD GSVTNTLENPGSLIKFTYKAKGGSGGSGGGSQVQLVESGGGLVQAGGS LRLSCAASRRSSRSWAMAWFRQAPGKEREFVAKISGDGRLTTYGDSVK GRFTISRDNAEYLVYLQMDSLKPEDTAVYYCAADDNYVTASWRSGPDY WGQGTQVTVSSKLAAALEHHHHHH
GDH-CaM	DVPLIPSQFAKAKSENFDKKVILSNLNKPHALLWGPDNQIWLTERATGK ILRVNPESGSVKTVFQVPEIVNDADGQNGLLGFAFHPDFKNNPYIYISGT FKNPKSTDKELPNQTIIRRYTYNKSTDTLEKPVDLLAGLPSSKDHQSGRL VIGPDQKIYYTIGDQGRNQLAYLFLPNQAQHTPTQQELNGKDYHTYMG KVLRLNLDGSIPKDNPSFNGVVSHIYTLGHRNPQGLAFTPNGKLLQSEQ GPNSDDEINLIVKGGNYGWPNVAGYKDDSGYAYANYSAAANKTIKDLA QNGVKVAAGVPVTKESEWTGKNFVPPLKTLYTVQDTYNYNDPTCGEMT YICWPTVAPSSAYVYKGGKKAITGWENTLLVPSLKRGVIFRIKLDPTYST TYDDAVPMFKSGSGGTEEQIAEFKEAFSLFDKDGDGTITTKELGTVMRS LGQNPTEAELQDMINEVDADGNGTIDFPEFLTMMARKMKDTDSEEEIR EAFRVFDKDGNGYISAAELRHVMTNLGEKLTDEEVDEMIREADIDGDG QVNYEEFVQMMTAGGSSGNRYRDVIASPDGNVLYVLTDTAGNVQKDD GSVTNTLENPGSLIKFTYKAKKLAAALEHHHHHH
FKBP-GDH-CaM	DVPLIPSQFAKAKSENFDKKVILSNLNKPHALLWGPDNQIWLTERATGK ILRVNPESGSVKTVFQVPEIVNDADGQNGLLGFAFHPDFKNNPYIYISGT FKNPKSTDKELPNQTIIRRYTYNKSTDTLEKPVDLLAGLPSSKDHQSGRL

	VIGPDQKIYYTIGDQGRNQLAYLFLPNQAQHTPTQQELNGKDYHTYMG KVLRLNLDGSIPKDNPSFNGVVSHIYTLGHRNPQGLAFTPNGKLLQSEQ GPNSDDEINLIVKGGNYGWPNVAGYKDDSGYAYANYSAAANKTIKDLA QNGVKVAAGVPVTKESEWTGKNFVPPLKTLYTVQDTYNYNDPTCGEMT YICWPTVAPSSAYVYKGGKKAITGWENTLLVPSLKRGVIFRIKLDPTYST TYDDAVPMFKSGCGGTEEQIAEFKEAFSLFDKDGDGTITTKELGTVMRS LGQNPTEAELQDMINEVDADGNGTIDFPEFLTMMARKMKDTDSEEEIR EAFRVFDKDGNGYISAAELRHVMTNLGEKLTDEEVDEMIREADIDGDG QVNYEEFVQMMTAGGSCGNRYRDVIASPDGNVLYVLTDTAGNVQKDD GSVTNTLENPGSLIKFTYKAKGGSGGGVQVETISPGDGRTFPKRGQTCV VHYTGMLEDGKKFDSSRDRNKPFKFMLGKQEVIRGWEEGVAQMSVGQ RAKLTISPDYAYGATGHPGIIPPHATLVFDVELLKLEKLAAALEHHHHHH
FRB-CaM BP	AHHHHHHSSGTRVAILWHEMWHEGLEEASRLYFGERNVKGMFEVLEP LHAMMERGPQTLKETSFNQAYGRDLMEAQEWCRKYMKSGNVKDLTQ AWDLYYHVFRRISGGSGGSGSGSGGSGGKRRWKKNFIAVSAANR
M13 CaM-BP	KRRWKKNFIAVSAANRFKKISSSGAL

Supplementary Table 2: Statistics of diffraction data and refinement.

**Data collection**	*nanoCLAMP8-VHH*	*nanoCLAMP3-VHH*
Wavelength (Å)[a]	0.95372	0.95373
Resolution (Highest Shell, Å)	59.05 – 1.83 (1.88 – 1.83)	48.38 – 2.9 (2.975 – 2.9)
Space group	C2221	I2
Cell constants (Å; º)	a=54.1, b=95.9, c=118.1; α=β=γ=90	a=173.2, b=144.0, c=181.8; α=90, β=94.3, γ=90
V_M	2.36	4.95
Total measurements	375031(21790)	1401844(70152)
Unique reflections	27424(1597)	98506(4830)
Average redundancy	13.7 (13.6)	14.2 (14.5)
I/σ	16.7 (3.5)	7.5 (0.7)
Completeness (%)	99.5 (96.5)	100.0 (100.0)
Rpim	0.036 (0.222)	0.086 (1.210)
CC1/2	0.999 (0.952)	0.994 (0.340)
**Refinement**		
Resolution (Highest Shell, Å)	1.83 (1.87 – 1.83)	2.9 (2.975 – 2.9)
R[b]	14.9(24.6)	19.5(34.0)
R_{free}[c]	20.2(42.6)	24.2(38.7)
rmsd bonds (Å) / angles (º)	0.026/2.049	0.021/2.057
B-factor deviation bonds / angles (Å²) main chain	1.826/2.781	0.949/1.835

side chain	3.874/5.405	2.421/4.204
Residues in Ramachandran Core (%)[d]	98.17	91.70
Protein atoms<br>solvent atoms<br>ligand atoms	2200<br>830<br>35	14529<br>3<br>245
Average B-factor (Å^2)	19	73
PDB accession code	7RG7	7RGA

[a]All data were collected at beamline MX1 or MX2 of the Australia Synchrotron (Melbourne, Australia)
[b]R is the R-factor = (Σ | F_O | - Σ | F_C |) / Σ | F_O |.
[c]R_{free} is the R-factor calculated using 5% of the data that were excluded from the refinement.
[d]Ramachandran core refers to the most favoured regions in the φ/ψ-Ramachandran plot
