# Supplementary material for: Design of a methotrexate-controlled chemical dimerization system and its use in bio-electronic devices
Source: Nat Commun. 2021 Dec 8;12:7137. doi: 10.1038/s41467-021-27184-w (PMC8654847; doi:10.1038/s41467-021-27184-w)
Supplement: Supplementary file 2 — Reporting Summary [file 41467_2021_27184_MOESM2_ESM.pdf]

nature portfolio

Corresponding author(s): Kirill Alexandrov

Last updated by author(s): Oct 21, 2021

Reporting Summary

Nature Portfolio wishes to improve the reproducibility of the work that we publish. This form provides structure for consistency and transparency in reporting. For further information on Nature Portfolio policies, see our Editorial Policies and the Editorial Policy Checklist.

Statistics

For all statistical analyses, confirm that the following items are present in the figure legend, table legend, main text, or Methods section.

n/a	Confirmed	
☐	☒	The exact sample size (n) for each experimental group/condition, given as a discrete number and unit of measurement
☐	☒	A statement on whether measurements were taken from distinct samples or whether the same sample was measured repeatedly
☒	☐	The statistical test(s) used AND whether they are one- or two-sided<br>*Only common tests should be described solely by name; describe more complex techniques in the Methods section.*
☒	☐	A description of all covariates tested
☒	☐	A description of any assumptions or corrections, such as tests of normality and adjustment for multiple comparisons
☐	☒	A full description of the statistical parameters including central tendency (e.g. means) or other basic estimates (e.g. regression coefficient) AND variation (e.g. standard deviation) or associated estimates of uncertainty (e.g. confidence intervals)
☒	☐	For null hypothesis testing, the test statistic (e.g. F, t, r) with confidence intervals, effect sizes, degrees of freedom and P value noted<br>*Give P values as exact values whenever suitable.*
☒	☐	For Bayesian analysis, information on the choice of priors and Markov chain Monte Carlo settings
☒	☐	For hierarchical and complex designs, identification of the appropriate level for tests and full reporting of outcomes
☒	☐	Estimates of effect sizes (e.g. Cohen's d, Pearson's r), indicating how they were calculated

Our web collection on statistics for biologists contains articles on many of the points above.

Software and code

Policy information about availability of computer code

Data collection	Microscale thermophoresis - M.Control software , GDH absorption assays - Cary WinUV Software, Electrochemical measurements- Digy-Ivy DY2116B software, HDX -DynamX v3.0 , X-ray data -REFMAC5 and COOT
Data analysis	Grafit 5.04, Prism 9.2

For manuscripts utilizing custom algorithms or software that are central to the research but not yet described in published literature, software must be made available to editors and reviewers. We strongly encourage code deposition in a community repository (e.g. GitHub). See the Nature Portfolio guidelines for submitting code & software for further information.

Data

Policy information about availability of data

All manuscripts must include a data availability statement. This statement should provide the following information, where applicable:

- Accession codes, unique identifiers, or web links for publicly available datasets
- A description of any restrictions on data availability
- For clinical datasets or third party data, please ensure that the statement adheres to our policy

the PDB accession codes for structures used in this manuscript are 3QXV, 3QXU, 2W1Q, 1DDS, 1AXW. The new structures presented in this manuscript in this manuscript are 7RG7 and 7RGA

Field-specific reporting

Please select the one below that is the best fit for your research. If you are not sure, read the appropriate sections before making your selection.

- [x] Life sciences
- [ ] Behavioural & social sciences
- [ ] Ecological, evolutionary & environmental sciences

For a reference copy of the document with all sections, see nature.com/documents/nr-reporting-summary-flat.pdf

Life sciences study design

All studies must disclose on these points even when the disclosure is negative.

Sample size	The sample size applies only to experiments on analysis of MTX concentration in patient's samples where 15 patient samples were chosen to cover the full clinical range of MTX . The number was chosen based on considerations of experimental effort required to perform the analysis and the number of samples as well as having at least 10 samples to represent the more challenging samples with <1micromolar concentrations
Data exclusions	no data was excluded from analysis
Replication	Analysis of MTX concentrations in titration experiments were considered as individual data sets and fitted as such with standard errors reflecting deviation from the theoretical fit. all experiments were successful. The same applies to MST experiments where all experiments were successful . Quantification of MTX in human serum was performed in technical triplicates and all experiments were successful. The analysis of the bio-electrode response to experimental conditions was performed using three independent electrodes for each data point. After optimization of the experimental set up 80% of the experiments were sucsesful.
Randomization	N/A
Blinding	Blinding was performed for the MTX quantification in patient's samples . Here samples cecieved form the pathology lab did not have any identification information and were independently analyzed using developed MTX assay. The reference data was unblinded after the quantification results were obtained and used to generate the correlation plot presented in figure 4F

Reporting for specific materials, systems and methods

We require information from authors about some types of materials, experimental systems and methods used in many studies. Here, indicate whether each material, system or method listed is relevant to your study. If you are not sure if a list item applies to your research, read the appropriate section before selecting a response.

Materials & experimental systems

n/a	Involved in the study	
☒	☐	Antibodies
☒	☐	Eukaryotic cell lines
☒	☐	Palaeontology and archaeology
☒	☐	Animals and other organisms
☒	☐	Human research participants
☒	☐	Clinical data
☒	☐	Dual use research of concern

Methods

n/a	Involved in the study	
☒	☐	ChIP-seq
☒	☐	Flow cytometry
☒	☐	MRI-based neuroimaging
